# The Elongator subunit Elp3 is a non-canonical tRNA acetyltransferase

**DOI:** 10.1038/s41467-019-08579-2

**Published:** 2019-02-07

**Authors:** Ting-Yu Lin, Nour El Hana Abbassi, Karol Zakrzewski, Andrzej Chramiec-Głąbik, Małgorzata Jemioła-Rzemińska, Jan Różycki, Sebastian Glatt

**Affiliations:** 1 Max Planck Laboratory, Malopolska Centre of Biotechnology (MCB), Jagiellonian University, Krakow, 30-387 Poland; 2 Postgraduate School of Molecular Medicine, Warsaw, 02-091 Poland; 3 Faculty of Biochemistry, Biophysics and Biotechnology, Jagiellonian University, Krakow, 30-387 Poland; 4 Malopolska Centre of Biotechnology (MCB), Jagiellonian University, Krakow, 30-387 Poland; 5 Bionanoscience and Biochemistry Laboratory, Malopolska Centre of Biotechnology (MCB), Jagiellonian University, Krakow, 30-387 Poland

## Abstract

The Elongator complex catalyzes posttranscriptional tRNA modifications by attaching carboxy-methyl (cm^5^) moieties to uridine bases located in the wobble position. The catalytic subunit Elp3 is highly conserved and harbors two individual subdomains, a radical *S*-adenosyl methionine (rSAM) and a lysine acetyltransferase (KAT) domain. The details of its modification reaction cycle and particularly the substrate specificity of its KAT domain remain elusive. Here, we present the co-crystal structure of bacterial Elp3 (DmcElp3) bound to an acetyl-CoA analog and compare it to the structure of a monomeric archaeal Elp3 from *Methanocaldococcus infernus* (MinElp3). Furthermore, we identify crucial active site residues, confirm the importance of the extended N-terminus for substrate recognition and uncover the specific induction of acetyl-CoA hydrolysis by different tRNA species. In summary, our results establish the clinically relevant Elongator subunit as a non-canonical acetyltransferase and genuine tRNA modification enzyme.

## Abstract (web summary)

Elp3 is the catalytic subunit of the eukaryotic Elongator complex that catalyzes posttranscriptional tRNA modifications. Here the authors present the crystal structures of an acetyl-CoA analog bound bacterial Elp3 and a monomeric archaeal Elp3 and show that Elp3 functions as a tRNA modification enzyme in all domains of life.

## Introduction

Over 170 different RNA base modifications are currently described^1^ and a majority is found in tRNA molecules of all three domains of life^2,3^. The functions of tRNA modifications can be separated into three main categories depending on the modified positions, either (i) stabilizing the structural integrity of the core tRNA fold^4^, (ii) contributing to the correct amino-acylation of respective tRNAs at the acceptor stem loop^5^ or (iii) enhancing the decoding potential and translation fidelity at the ribosome^6,7^. The latter group of tRNA modifications is mostly found around the anticodon stem loop (ASL), particularly at the so-called “hot spot” positions 34 and 37^2,7,8^. As modifications in this region can provide additional chemical bonds between the ASL and its cognate and near-cognate codons during the ribosomal decoding process, they are crucial for fine-tuning translation elongation^8–11^ and co-translational folding dynamics^12,13^. In agreement, the lack of certain uridine modifications in the wobble position (U_34_), such as 5-methoxycarbonylmethyl (mcm^5^), 5-carbamoylmethyl (ncm^5^) and 5-methoxy-carbonyl-methyl-2-thio (mcm^5^s^2^), were shown to induce cellular stress^14^, increase intracellular protein aggregation and disturb proteome homeostasis^15,16^.

The eukaryotic Elongator complex was initially described as a transcription-related elongation factor due to its association with hyper-phosphorylated RNA Polymerase II^17^ and the predicted presence of a potential histone/lysine acetyltransferase (KAT) domain in its catalytic Elp3 subunit^18^. Although some recent reports still follow that initial hypothesis, an increasing number of studies supports the idea that Elongator in fact represents a genuine tRNA modification enzyme, which catalyzes the cm^5^U_34_ modification, representing the first step in a cascade leading to different types of U_34_ modifications^19–22^. The cm^5^ moiety can be subsequently methylated by the methyl transferase Trm9 resulting in mcm^5^U_34_ ref. ^23^. In three yeast tRNAs, namely tRNA^Glu^_UUC_, tRNA^Gln^_UUG_, and tRNA^Lys^_UUU_, this “primal” Elongator modification is succeeded by an additional thiolation^24^ leading to mcm^5^s^2^U_34_ or is converted into ncm^5^U_34_ by a yet unknown mechanism in other tRNA species^25^.

The fully assembled eukaryotic complex consists of two copies of each of its six subunits (Elp1-6), which are arranged in two-independent modules, the catalytic Elp123 and the associated Elp456 sub-complexes^26–28^. All six Elongator subunits are highly conserved among eukaryotes and the Elp3 subunit is even found in all three domains of life, including all archaea and some bacterial clades^20^. Elp3 acts as the catalytic subunit, but the loss of any of the six subunits results in hypo-modified U_34_ tRNAs in yeast^19,29^, indicating that the complete integrity of the complex is important for its function. The key role of Elongator in maintaining the stringent homeostasis of the cellular proteome explains the pleotropic phenotypes associated with Elp3 deficiency, including neurogenesis^30^, DNA repair^31^, exocytosis^32^, genome demethylation^33^, protein acetylation^34,35^, mitochondria dysfunction^36^, and tRNA modification^22^. Moreover, patient-derived mutations and deficiencies in different Elongator subunits are associated with severe human diseases^37,38^, such as cancer^39^ and neurodegenerative diseases^40^, including familial dysautonomia^41^, amyotrophic lateral sclerosis^42^, intellectual disabilities^43^, and ataxia^44^.

Over the last decade, significant progress has been made towards an understanding of the structural and functional rationale behind the modification reaction conducted by the catalytically active Elp3 subunit^20,21,45,46^. We previously determined the high resolution crystal structure of the bacterial Elp3 homologue from *Dehalococcoides mccartyi* (DmcElp3), revealing the tight interplay between the involved radical *S*-adenosyl methionine (rSAM) and KAT domains. The rSAM domain of Elp3 is highly similar to RlmN^47^, which also contains a Fe–S cluster and methylates rRNAs and tRNAs using its reductive SAM cleavage activity^48^. The C-terminal KAT domain is highly similar to the known superfamily of GCN5-like acetyl transferases^49^, which bind linear peptides and acetylate target lysines via their acetyl-CoA hydrolysis activity^50^. The two domains form a large domain interface, which blocks the canonical KAT peptide-binding site, and a partially disordered central linker region coordinates a Zn-ion in between rSAM and KAT. The two domains form a cleft that contains several highly conserved basic residues, which bind and accommodate the tRNA anticodon stem loop (ASL). The current model of a radical based Elp3-mediated cm^5^-modification reaction is proposed as follows (i) Elp3 recruits SAM and cleaves it to generate a 5’deoxyadenosine radical (5’-dA) in the rSAM domain, (ii) Elp3 hydrolyzes acetyl-CoA in the KAT domain and (iii) an acetyl radical is formed by the products of the two domains and (iv) is transferred onto the C5 position of U_34_ in the bound tRNA molecule.

In comparison to the overwhelming amount of recently published phenotypical and clinical studies on the Elongator complex, the number of available structural and biochemical data remain minuscule. However, without a deeper mechanistic knowledge of the underlying mechanisms in different organisms, the genuine cellular function of Elongator and the details of the reaction cycle and its intermediates remain elusive and highly speculative. Here, we present the structure of DmcElp3 bound to an acetyl-CoA derivative that reveals ligand coordination and allows the precise identification of several active site residues. In addition, we present the crystal structure of monomeric Elp3 from archaea and compare it to the previously known dimeric bacterial Elp3 structure. Furthermore, we are able to measure acetyl-CoA hydrolysis rates and tRNA binding activities for different Elp3 homologues. Last but not least, we provide biochemical evidence that specific tRNAs, but neither histone or tubulin peptides nor other nucleic acids, act as the exclusive trigger for acetyl-CoA hydrolysis in bacterial, archaeal and eukaryotic Elp3s. Our results define Elongator and its Elp3 subunit as a genuine tRNA editing enzyme in all three domains of life and resolve the ongoing debate about a direct involvement of Elongator in any cellular activity other than tRNA modification.

## Results

### DmcElp3 binds acetyl-CoA like other KAT domain proteins

**Fig. 1:**
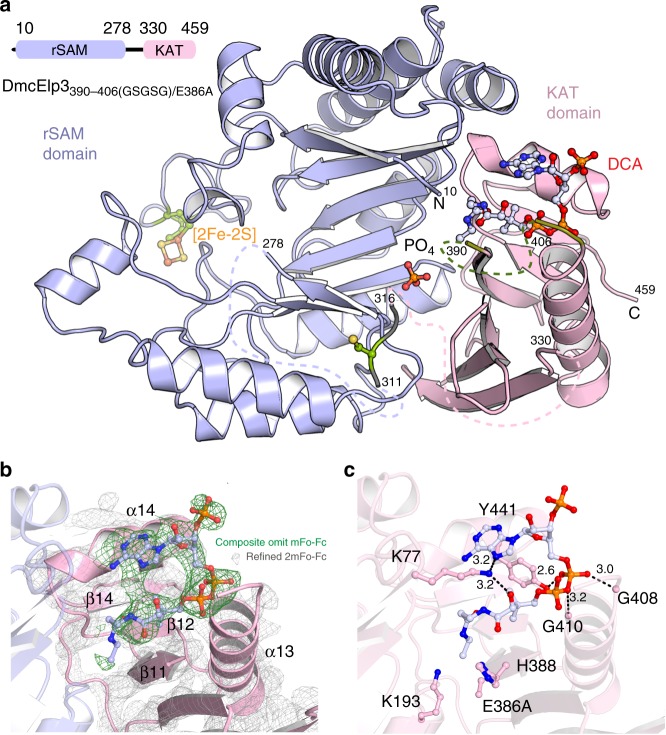
The crystal structure of DCA bound to DmcElp3_390–406(GSGSG)/E386A_. **a** Overall structure of DmcElp3-DCA in cartoon representation. The rSAM domain (blue), KAT domain (pink), and acetyl-CoA blocking loop (olive) are highlighted. Disordered loops are shown using dotted lines. Fe–S cluster binding and zinc binding residues are highlighted in green. Fe–S cluster (orange and yellow spheres) and PO_4_ (orange and red spheres) are highlighted. Respective amino acid numbers are indicated. **b** The Fo–Fc simulated annealing omit map (green) of DCA and the refined 2Fo–Fc map (gray) are shown at 1.5 σ in the binding pocket of DmcElp3_390–406(GSGSG)/E386A_. The model of DmcElp3 is shown in cartoon and the located DCA molecule in ball and sticks representations. **c** The interaction of DCA and the residues residing in the binding pocket of DmcElp3_390–406(GSGSG)/E386A_. Hydrogen bonds are highlighted in dotted lines while bond lengths were analyzed using LigPlot. All involved residues and distance are indicated

We have previously determined the crystal structure of Elp3 from *Dehalococcoides mccartyi* and identified the potential acetyl-CoA binding pocket and a specific blocking loop that seems to functionally link acetyl-CoA- and tRNA-binding^21^. Furthermore, we found that several potent key residues residing in the acetyl-CoA binding pocket (e.g. K77, K193, E386, and Y441) are conserved among various Elp3s and showed that the equivalent single amino acid substitutions in yeast lead to Elongator loss-of-function phenotypes. To obtain detailed structural insights into acetyl-CoA coordination, we combined the respective blocking loop deletion (DmcElp3_390–406(GSGSG)_) with additional active site mutations (e.g. E386A, H388A, R411T) for co-crystallization trials in the presence of acetyl-CoA and different acetyl-CoA analogues. We successfully co-crystallized DmcElp3_390–406(GSGSG)/E386A_ (Supplementary Fig. 1a) in complex with desulfo-CoA (DmcElp3-DCA) using similar crystallization conditions as for apo DmcElp3. We solved and refined the structure at 2.7 Å resolution using DmcElp3 (PDB ID 5L7L) as search model for molecular replacement (Fig. 1a and Table 1). The overall structure of DmcElp3-DCA closely matches the structure of DmcElp3 (Supplementary Fig. 1b), indicating that ligand binding has very little or no influence on the structural arrangement of the two domains or the Fe–S cluster mediated rSAM dimerization. This observation is consistent with our previous results from SAXS analyses performed in the presence and absence of acetyl-CoA^21^. The density of DCA is clearly visible in initial, refined and simulated annealing composite omit maps and allowed its unambiguous placement (Fig. 1b). DCA is structurally similar to acetyl-CoA but lacks both, the acetyl group as well as the thiol-moiety, and therefore represents a post-cleavage product analogue. DCA binds to the KAT domain of DmcElp3 in a similar way as acetyl-CoA is coordinated by the active site of GCN5 from *Tetrahymena* (Supplementary Fig. 1b). In detail, the two phosphate groups of DCA are in close proximity to the glycine residues residing at the start of the blocking loop. The rest of DCA, including pantothenic acid and β-mercaptoethylamine, are pointing deeper into the predicted binding pocket. Furthermore, previously identified conserved residues, including K77, K193, and Y441, are involved in ligand binding and in close proximity to DCA (Fig. 1c and Supplementary Fig. 1c). The β-mercaptoethylamine part of DCA was not clearly resolved in our densities, presumably because this part of the ligand is flexible in the absence of the missing tRNA substrate. Considering the observation that DCA stays bound even in the absence of the transferable acetyl group, we conclude that the cleavage products stay associated with the KAT domain until the next round of modification is initiated.

**Table 1:** Data collection, phasing and refinement statistics

	DmcElp3 390–406_GSGSG_/E386A DCA PDB ID 6IA6	MinElp3 Δ1–19 PDB ID 6IA8	MinElp3 Δ1–46 PDB ID 6IAZ	MinElp3 Δ1–54 PDB ID 6IAD
*Data collection*				
Space group	C222_1_	P4_1_2_1_2	P4_1_2_1_2	P4_1_2_1_2
*Cell dimensions*				
*a*, *b*, *c* (Å)	73.56, 160.74, 93.00	63.44, 63.44, 250.22	63.35, 63.35, 250.06	62.97, 62.97, 248.25
*α*, *β*, *γ* (°)	90, 90, 90	90, 90, 90	90, 90, 90	90, 90, 90
Wavelength	0.9184	1.0332	0.9184	0.9184
Resolution (Å)^†^	50–2.7(2.77–2.7)	50.0–1.9 (1.95–1.9)	50–1.9 (1.95–1.9)	50–2.05 (2.10–2.05)
*R*_meas_ (%)	14.1 (209.9)	6.8 (158.2)	6.8 (99.7)	10.5 (162.9)
*I/σ*(*I*)	9.75 (0.81)	33.38 (1.73)	19.34 (1.82)	16.08 (1.76)
*CC*_1/2_	0.99 (0.29)	1.00 (0.59)	0.99 (0.62)	0.99 (0.62)
Completeness (%)	99.0 (98.1)	98.3 (83.4)	99.8 (98.0)	99.8 (98.1)
Redundancy	4.4 (4.0)	24.1 (16.42)	10.4 (8.3)	12.7 (12.8)
*Refinement*				
Resolution (Å)	43.3–2.7	44.9–1.9	34.8–1.9	29.4–2.05
No. of reflections	15,372	41,022	41,281	32,466
*R*_work_/*R*_free_	22.3/26.9	18.4/20.7	19.5/21.7	19.7/22.3
*No. of atoms*				
Protein	3017	3487	3476	3494
Ligand/ion	56	35	30	20
Water	0	124	163	109
*B factors*				
Protein	83.7	50.4	47.1	51.1
Ligand/ion	125.4	87.6	88.5	93.1
Water	0	48.3	47.4	49.6
*r.m.s deviations*				
Bond lengths (Å)	0.002	0.009	0.005	0.002
Bond angles (°)	0.552	0.847	0.730	0.635

Values in parentheses are for highest-resolution shell

^†^Resolution limits according to *I*/*σ* of 2 are 2.95 Å for DmcElp3_390–406(GSGSG)/E386A_DCA, 1.9 Å for MinElp3_Δ1–19_, 1.9 Å for MinElp3_Δ1–46_, 2.1 Å for MinElp3_Δ1–54_

### Structural comparison between bacterial and archaeal Elp3

DmcElp3 represents a rare example of bacterial Elp3s, but shares high sequence similarity with archaeal Elp3s, which also lack genes for all the other five eukaryotic Elongator subunits. Although DmcElp3 and *Methanocaldococcus infernus* Elp3 (MinElp3) share 50% sequence identity, MinElp3 harbors an extended N-terminus (aa 1–75). This flexible region enriched in basic residues is present in most archaeal and eukaryotic Elp3 sequences, but completely absent from DmcElp3^21^. To gain further structural and functional insights into archaeal and more closely related eukaryotic Elp3s, we expressed and purified recombinant full length MinElp3 under aerobic conditions. Unlike DmcElp3, which shows two discrete oligomeric states^21^, full length MinElp3 exclusively forms monomers. We further generated five different N-terminally truncated versions (MinElp3_Δ1–19_, MinElp3_Δ1–41_, MinElp3_Δ1–46_, MinElp3_Δ1–54_, MinElp3_Δ1–68_) to facilitate crystallization. In comparison to the full length protein, all five truncations exhibited two discrete concentration-independent oligomeric states in size exclusion chromatography, namely monomers and dimers (Supplementary Fig. 2a). All purified MinElp3 samples appeared slightly pink, suggesting the presence of an Fe–S cluster in a minority of monomeric and dimeric MinElp3 molecules even under aerobic purification conditions. Moreover, the Fe–S cluster loop (like in DmcElp3) directly contributes to the dimer formation of truncated MinElp3 (Supplementary Fig. 2a). Notably, the monomeric forms of truncated MinElp3 elute earlier from the gel filtration column and therefore appear to be larger than expected. We confirmed their theoretical masses by SDS-PAGE and light scattering (RALS and LALS) measurements and speculate that the N-terminus could also be involved in the compaction of the bacterial and archaeal Elp3 proteins.

**Fig. 2:**
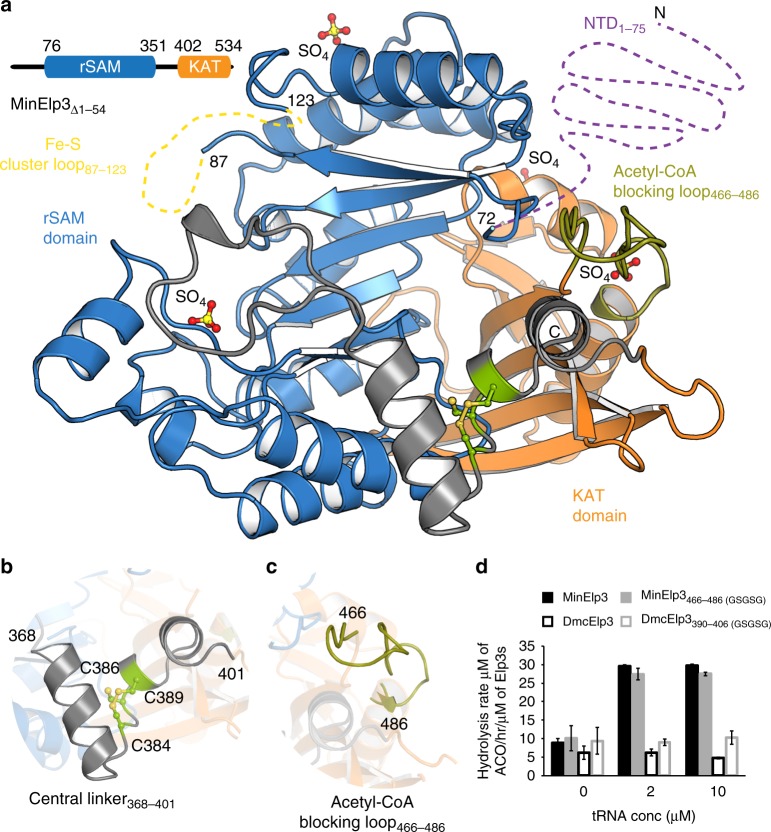
The crystal structure of MinElp3_Δ1–54_. **a** Overall structure of MinElp3_Δ1–54_ in cartoon representation. The rSAM domain (blue), KAT domain (orange), acetyl-CoA blocking loop (olive), and central linker region (gray) are highlighted. Disordered loops are shown using dotted lines. Cysteines in the central linker region are highlighted in green and SO_4_ is shown as orange and red spheres. The respective amino acid numbers are indicated. **b** Close-up view of the central linker region that connects rSAM and KAT domains. The three cysteine residues involved in disulfide bond formation are indicated (yellow). **c** Close-up view of the acetyl-CoA blocking loop (olive). **d** The acetyl-CoA hydrolysis rates of MinElp3, MinElp3_466–486(GSGSG)_, DmcElp3 and DmcElp3_390–406(GSGSG)_ in the absence and presence of endogenous yeast bulk tRNA (*Δelp3;* UMY2916). Data represent mean ± standard error of the mean (SEM). Source data are provided as a Source Data file

We used full length and truncated versions of purified MinElp3 in crystallization trials and obtained well-diffracting crystals for monomeric MinElp3_Δ1–19_, MinElp3_Δ1–46_, and MinElp3_Δ1–54_. We solved their structures using a poly-alanine model of DmcElp3 via molecular replacement at 1.9 Å, 1.9 Å, and 2.1 Å resolution, respectively (Fig. 2a and Table 1). Although all three versions contained various lengths of the N-terminus and also showed no signs of degradation, we could observe interpretable electron density only starting from residue V72 in all collected and refined datasets. Therefore, we believe that the N-terminus adopts a highly flexible conformation in the absence of substrate tRNAs. The overall structure and domain arrangement and interface between the rSAM and KAT domains of MinElp3 are almost identical to DmcElp3 (Supplementary Fig. 2b,c). The main difference relates to the Fe–S cluster loop region (aa 87–123) and the Fe–S cluster itself, which were both completely unstructured in all monomeric MinElp3s (Fig. 2a and Supplementary Fig. 2d). We previously identified a Zn-binding motif in the central linker region between the two domains that is coordinated by three cysteine residues, which are strictly conserved in bacterial and archaeal but missing in eukaryotic Elp3s (Fig. 2b)^21^. In our MinElp3 structures, we could not locate any bound Zn-ions and instead observed the formation of a disulfide bond between C384 and C389. Nevertheless, the equivalent sequence still obtains an almost identical conformation, indicating that zinc might simply contribute structural integrity in anaerobic bacteria. In addition, we observed that the acetyl-CoA blocking loop (aa 466–486) is positioned outside of the acetyl-CoA binding pocket, interacting with the last visible residues of the N-terminus and also contacting a neighboring symmetry mate (Fig. 2c and Supplementary Fig. 2e). Using our DmcElp3-DCA structure as a reference model, we are able to show that acetyl-CoA would also fit seamlessly into the active site of MinElp3, indicating an almost identical ligand coordination and active site organization. In summary, bacterial and archaeal Elp3 proteins do not only share high sequence conservation (Supplementary Fig. 2d and Supplementary Fig. 3a), but also adopt an almost identical overall domain arrangement indicating that they represent examples of the genuine structure of all Elp3 proteins.

### Functional comparison between bacterial and archaeal Elp3

**Fig. 3:**
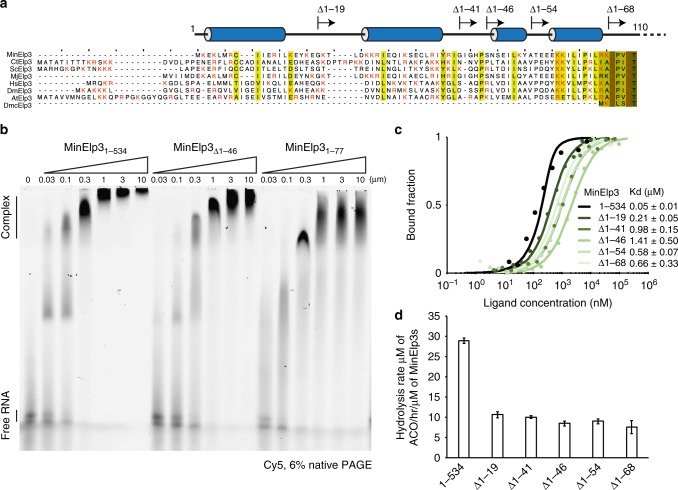
N-terminus features of MinElp3 in tRNA binding and acetyl-CoA hydrolysis. **a** Sequence alignment of N-termini in selected Elp3 proteins. *D. mccartyi* (DmcElp3) (YP307690), *M. infernus* (MinElp3) (YP003616086), *M. jannaschii* (MjElp3) (AAB99138), *S. cerevisiae* (ScElp3) (NP015239), *D. melanogaster* (DmElp3) (NP608834), *A. thaliana* (AtElp3) (NP568725), *H. sapiens* (HsElp3) (NP060561), *C. thermophilum* (CtElp3) (XP_006697164.1). Relatively conserved residues are labeled in gradient green depending on the conservation scores. Basic residues are labeled in red. The predicted secondary structure elements of MinElp3 are indicated on top of the aligned sequences. The individual start sites of the truncated N-terminal constructs are indicated. **b** EMSA analyses of MinElp3s binding to yeast tRNA^Ser^_UGA_ (Cy5-labeled). The samples were resolved in a 6% native PAGE and visualized using a Typhoon scanner. Protein concentrations are given and positions of free tRNA and protein-tRNA complex are labeled. **c** MST analyses of MinElp3s binding to (Dmc)tRNA^Glu^_UUC_ and the *K*_d_s are indicated, *n* = 3. **d** Acetyl-CoA hydrolysis reaction of different MinElp3 N-terminus truncations in the presence of endogenous yeast bulk tRNA (*Δelp3;* UMY2916), *n* = 3. Data represent mean ± SEM. Source data are provided as a Source Data file

It was recently shown that the acetyl-CoA hydrolysis activity of MinElp3 can be induced by the presence of in vitro transcribed and truncated tRNAs and that this trigger does not require the presence of an Fe–S cluster^20^. We compared the acetyl-CoA hydrolysis rates of DmcElp3 and MinElp3 using purified endogenous bulk tRNAs from an Elp3-depleted yeast strain, which carries naturally occurring modifications. Both proteins exhibited low intrinsic basal hydrolysis rates in the absence of tRNA, but MinElp3 as well as its mutated form lacking the acetyl-CoA blocking loop (MinElp3_466–486(GSGSG)_) showed robust acetyl-CoA hydrolysis activity after addition of bulk tRNA. Furthermore, we confirmed that MinElp3 binds to acetyl-CoA (*K*_d_ 135.4 ± 55.3 μM) and that deleting the acetyl-CoA blocking loop does not have significant effects on its affinity to acetyl-CoA in the absence of tRNA (*K*_d_ 107.7 ± 55.8 μM; Supplementary Fig. 3b). DmcElp3, on the other hand, showed ~5-times reduced-induced hydrolysis activity, which remained similarly weak after deletion of the acetyl-CoA blocking loop region (DmcElp3_390–406(GSGSG)_; Fig. 2d). The varying levels of inducibility correlate well with differences in the binding affinities of MinElp3 (*K*_d_ 50 ± 10 nM) and DmcElp3 (*K*_d_ ~1410 ± 230 nM) towards Cy5-labeled in vitro transcribed tRNA(Dmc)tRNA^Glu^_UUC_(CCA) (Supplementary Fig. 3c, d). As described above, the flexible N-terminus shows the biggest sequence variation among all known Elp3 proteins and also represents the main difference between MinElp3 and DmcElp3 sequences (Fig. 3a). Therefore, we analyzed if the extended flexible N-terminus of MinElp3 could account for the differences in tRNA binding affinity using electro-mobility shift assay (EMSA) and microscale thermophoresis (MST). We observed strong interaction of the purified MinElp3 N-terminus (MinElp3_1-77_) towards (Dmc)tRNA^Glu^_UUC_ and confirmed decreased affinities for N-terminally truncated MinElp3 proteins (Fig. 3b). We have designed and purified truncated versions sampling the whole N-terminus based on secondary structure predictions with the aim of conserving potential helices and referring stability (Fig. 3a). The first 45 residues seem to have a particularly strong contribution to the binding and deletion of the complete N-terminus resulted in affinities comparable to DmcElp3 (Fig. 3c). The decrease in tRNA affinities correlates well with a strong decrease in tRNA-induced acetyl-CoA hydrolysis activity of MinElp3, thereby confirming the specificity of the induction and emphasizing the contribution of Elp3 N-termini to tRNA binding and modification activity (Fig. 3d). As the N-terminal regions of Elp3 proteins are evolutionary very weakly conserved, we were curious if exchanging these sequences between species would affect the functionality. Strikingly, replacing the N-terminus of MinElp3 (aa 1–69) with the respective yeast (aa 1–95) or human (aa 1–78) sequences restored the tRNA binding affinity of N-terminally truncated MinElp3 (Supplementary Fig. 3d). As the tRNA-induced acetyl-CoA hydrolysis rates remained unchanged in these chimeric proteins (Supplementary Fig. 3e), it seems that although tRNA recognition is conserved, species-specific mechanisms is required to trigger the KAT activity.

### The acetyl-CoA hydrolysis of Elp3 is tRNA dependent

**Fig. 4:**
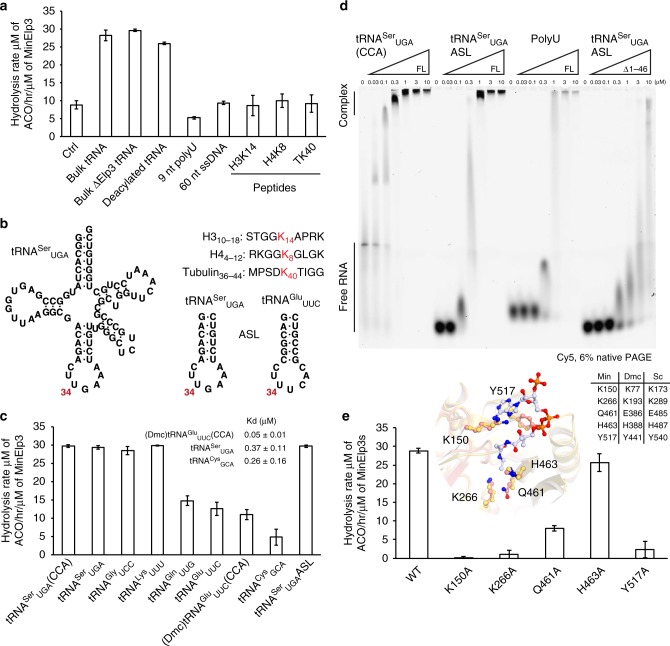
Substrate specificity feature of MinElp3 to activate acetyl-CoA hydrolysis. **a** Acetyl-CoA hydrolysis reactions of MinElp3 in the presence of different substrates, *n* = 3. **b** Chemical structure illustrations of tRNA and ASLs. Sequences of used peptides are given and modifiable lysine (K) residues are highlighted. **c** Acetyl-CoA hydrolysis reactions of MinElp3 in the presence of different tRNA substrates, *n* = 3 **d** EMSA analyses of MinElp3s binding to different nucleic acid substrates. Protein concentrations are given and positions of free tRNA and protein-tRNA complex are labeled. **e** Acetyl-CoA hydrolysis reactions of different MinElp3 point mutations in the presence of endogenous yeast bulk tRNA (*Δelp3;* UMY2916), *n* = 3. Superposition of highlighted residues in the acetyl-CoA binding pocket from MinElp3 (light pink) and DmcElp3 (yellow). DCA is shown in ball and sticks representation. The equivalent residue numbers in different Elp3s are indicated. Data represent mean ± SEM. Source data are provided as a Source Data file

Next, we tested whether other nucleic acids or the absence of charged amino acids could play a role for Elp3 activation. Both, bulk tRNA from wild yeast strains carrying endogenous levels of Elongator modifications, as well as de-acylated bulk tRNAs, efficiently activated acetyl-CoA hydrolysis of MinElp3 (Fig. 4a and Supplementary Fig. 4a, b). In contrast, neither polyU RNA nor single stranded DNA showed any detectable induction of hydrolysis rates. It has been an ongoing debate for many years, whether Elp3 could also catalyze the canonical acetylation of lysine residues in peptides and proteins. Therefore, we synthesized and tested lysine-containing histone (H3_10–18_ and H4_4–12_) and tubulin (Tubulin_36–44_) peptides, which have previously been suggested^18,51^ as potential Elongator substrates and shown to be suitable GCN5 substrates^52–54^ (Fig. 4b). Strikingly, neither histone nor tubulin peptides are able to bind archaeal MinElp3 (Supplementary Fig. 4c) nor trigger its acetyl-CoA hydrolysis reaction (Fig. 4a). Although in our hands all tested eukaryotic Elp3 proteins are highly unstable and insoluble in the absence of Elp1 and Elp2, we managed to obtain pure and homogenous KAT domain from *Chaetomium thermophilum* (CtElp3_435–574_), a thermophilic yeast strain^55^. All employed peptides failed to induce the enzymatic activity of this isolated KAT domain from this eukaryotic Elp3, even though the predicted peptide-binding site is principally exposed due to the absence of the rSAM domain (Supplementary Fig. 4d, e). Elp3 requires both domains to bind tRNAs to activate acetyl-CoA hydrolysis, explaining the observation that bulk tRNA also failed to induce the enzymatic activity of this eukaryotic Elp3 KAT domain.

### The tRNA substrate selectivity of Elp3

We subsequently analyzed whether any tRNA might trigger the acetyl-CoA hydrolysis of Elp3 or if the protein is able to select for suitable tRNA substrates. We initially noticed no binding preference for different in vitro transcribed tRNAs as the affinities for tRNA^Cys^_GCA_, tRNA^Ser^_UGA_, and tRNA^Glu^_UUC_ are in a comparable low nanomolar affinity range (Fig. 4c and Supplementary Fig. 5a). Therefore, MinElp3 binds equally well to unmodifiable (tRNA^Cys^_GCA_) and modifiable (tRNA^Ser^_UGA_/tRNA^Glu^_UUC_) tRNAs and is not capable of specifically recognizing the respective RNA base present at position 34. We subsequently tested several in vitro transcribed tRNAs, including tRNA^Lys^_UUU_, tRNA^Ser^_UGA_, tRNA^Gly^_UCC_, and tRNA^Cys^_GCA_, for their potential to induce acetyl-CoA hydrolysis. Whereas, tRNA^Ser^_UGA_ and tRNA^Gly^_UCC_ triggered the hydrolysis as well as endogenous bulk tRNA, we did not observe induction using tRNA^Cys^_GCA_, which harbors G_34_ (Fig. 4c). It appears that Elp3 shows broad specificity for tRNA binding, but strictly discriminates for modifiable U_34_-tRNAs at the step of acetyl-CoA hydrolysis. In addition, Elp3 seems to work independently from priming modification pathways, as endogenous fully decorated and in vitro transcribed “naked” tRNAs show an identical potential to induce its activity.

Next, we analyzed whether Elp3 discriminates between different modifiable tRNAs as cm^5^U_34_ primes for three downstream cascades, namely mcm^5^U_34_-, ncm^5^U_34_- and mcm^5^s^2^U_34_. Therefore, we tested tRNA^Glu^_UUC_, tRNA^Gln^_UUG_ and tRNA^Lys^_UUU_ all harboring mcm^5^s^2^U_34_ and an invariant U_35_ on their ability to induce acetyl-CoA activity. Strikingly, we observed regular levels of activity for tRNA^Lys^_UUU_, but only intermediate activities for tRNA^Glu^_UUC_, tRNA^Gln^_UUG_ and (Dmc)tRNA^Glu^_UUC_ (Fig. 4c). Initially, we were curious, if U_35_ next to U_34_ could cause a decreased acetyl-CoA hydrolysis activity, which is somehow restored in for tRNA^Lys^_UUU._ As the substitution of U_35_ to either purine (G_35_) or pyrimidine (C_35_) in yeast tRNA^Glu^ did not elevate its ability to induce the activity of MinElp3 (Supplementary Fig. 5b), we conclude that other determinants in tRNA^Glu^_UUC_ and tRNA^Gln^_UUG_ must be causative for their decreased activity.

We previously have shown that DmcElp3 not only uses its central basic cleft to bind the ASL of a tRNA, but also employs other basic residues residing in the N-terminus to make additional contacts around the D-loop of tRNAs^21^. To investigate whether these additional contacts are required for the enzymatic activation in MinElp3, we tested whether the ASL (derived from tRNA^Ser^_UGA_) is sufficient to activate Elp3. We observed that the ASL by itself is not only sufficient to bind to full length MinElp3, but also induces enzymatic activity at a comparable level as full length tRNA^Ser^_UGA_ (Fig. 4c, d). These observations are in line with a report that MinElp3 can modify tRNA^Arg^ that misses D- and T-loop^20^. We have previously reported that the ASL of (Dmc)tRNA^Glu^_UUC_ is not sufficient to bind to bacterial DmcElp3. In agreement with this data and the described importance of the extended N-terminus in MinElp3, we observed that the central basic cleft of MinElp3 (Supplementary Fig. 5c) is not sufficient for efficient ASL binding. The affinity between the truncated MinElp3_Δ1–46_ protein and the ASL alone are comparable to the one of an unspecific polyU sequence towards full length MinElp3 (Fig. 4d). As the presence of a 3’CCA sequence, added during the maturation of tRNAs, increases the affinity of full length MinElp3 for different tRNAs (Supplementary Fig. 5a), we speculate that the extended N-terminus might be involved in both, the recognition of the ASL and the 3’CCA regions.

### Identification of active site residues in the KAT domain

To identify the functional role of individual amino acids in the acetyl-CoA binding pocket of Elp3, we introduced several structure-guided mutations in MinElp3 (K150A, K266A, Q461A, H463A, and Y517A) and tested them for acetyl-CoA hydrolysis activity (Fig. 4e). Among these variants, only H463A still showed acetyl-CoA hydrolysis activity, whereas all remaining MinElp3 mutations (K150A, K266A, Q461A, and Y517A) showed strongly reduced or completely diminished tRNA-induced acetyl-CoA hydrolysis activity. The reduced hydrolysis activity of the affected mutants is not caused by alteration in their tRNA binding (Supplementary Fig. 6a), but reduced acetyl-CoA binding for K266A, Q461A, and Y517A. As K150A shows normal tRNA and acetyl-CoA binding, we believe that it might be directly involved the hydrolysis reaction (Supplementary Fig. 6b). Our previous in vivo analyses of the equivalent positions in yeast Elp3 (ScElp3) based on the apo structure of DmcElp3 confirmed the functional relevance of these residues for the eukaryotic Elongator complex^21^. To provide a complete mechanistic picture of the active site, we have undertaken major efforts to co-crystalize different Elp3 proteins in complex with various tRNA sequences, but to date have not succeeded in obtaining analyzable crystals. Future structural studies will take advantage of the identified mutants and use different combinations of ASLs and acetyl-CoA derivatives to lock Elp3 in different reaction intermediates.

## Discussion

Catalytic reactions and regulatory mechanisms of tRNA modification enzymes are diverse and complex^7^. The enzymatically active Elp3 subunit of Elongator contains two functional domains, utilizes multiple cofactors, and conducts a unique uridine base modification specifically found only at the wobble base position of tRNAs. Although, all known Elp3s share high sequence similarity, eukaryotic Elp3s are embedded in the large Elongator complex and the modification reaction requires five other Elongator subunits and several regulatory factors^56,57^. Nevertheless, it was recently shown that (m)cm^5^U_34_ is present in tRNAs from *H. volcanii* indicating that archaeal Elp3s must be able to conduct a very similar modification reaction as the fully assembled eukaryotic Elongator complex^58^. In this study, we provide a comprehensive structural and functional comparison of bacterial, archaeal and eukaryotic Elp3 proteins and their KAT domains.

**Fig. 5:**
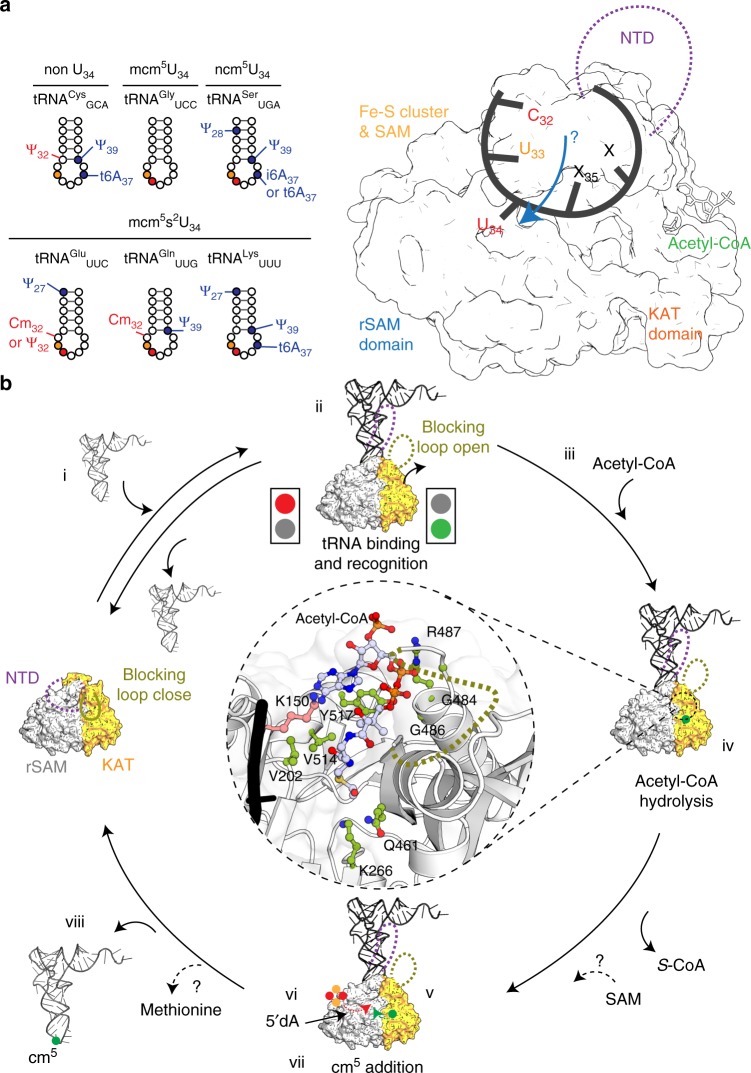
Proposed structural rearrangements of Elp3 during tRNA modification. **a** (left) Experimentally proven modifications in ASLs of different types of tRNAs. U_34_ is shown as red circles and the invariable U_33_ is shown as orange circles. Most common modifications are indicated (blue) while the modifications at position 32 of tRNA^Glu^_UUC_, tRNA^Gln^_UUG_, and tRNA^Cys^_GCA_ are labeled in red. Ψ: pseudouridine; Cm: 2-*O*-methylcytidine; i6A: N6-isopentenyladenosine; t6A: N6-threonylcarbamoyladenosine. (right) The proposed influence of priming modification on the cm^5^ modification mechanism at U_34_ (blue arrow). **b** Schematic overview of the individual sequential steps in the Elp3-mediated tRNA modification cycle. The N-terminus could change its location upon tRNA binding that regulates the position of acetyl-CoA blocking loop. The acetyl-CoA hydrolysis responsible residues (pink) and acetyl-CoA binding residues (green) are indicated in the inset. SAM is required for the catalytic reaction; however, the timing of SAM recruitment and release of the methionine by-product are still not clear (indicated by question marks)

Our results clearly illustrate that Elp3 can recognize and respond to in vitro transcribed tRNA substrates as well as to endogenous bulk tRNAs from yeast. We also demonstrate that a synthesized ASL can induce enzyme activity, whereas other tRNA sequences (e.g. tRNA^Glu^_UUC_, tRNA^Gln^_UUG_, (Dmc)tRNA^Glu^_UUC_, and tRNA^Cys^_GCA_) show reduced or no activity at all. These data indicate that specific bases in the ASL of tRNA are necessary to activate Elp3 and that for certain modifiable tRNAs additional modification might also be necessary for full activation (Supplementary Fig. 7). Thus, we searched the MODOMICS database^1^ of experimentally proven tRNA modifications in eukaryotic organisms and found that there are two specific modifications in the ASL of tRNA^Glu^_UUC_ and tRNA^Gln^_UUG_, which are not found in tRNA^Lys^_UUU_ or other modifiable tRNAs (Fig. 5a). They carry a pyrimidine modification at position 32 (either Cm_32_ or pseudo-uridine (Ψ_32_)), which is absent in the used in vitro transcribed tRNAs. The lack of Cm_32_ is known to affect the anticodon stem loop structure and function^7,59^, and thereby could explain the reduced activity of Elp3 observed with these two tRNA species. Several studies have reported on the cross-talk and interdependency of tRNA modification networks^60^ and the link between these modifications and Elp3 activation needs to be investigated in the future.

By combining all available biochemical and structural data, we can conclude a potential mechanism of regulation and a sequential order of the Elp3 reaction cycle (Fig. 5b). (i) Modifiable and non-modifiable tRNAs bind to Elp3 with similar affinity; (ii) the N-terminus of Elp3 checks for the presence of 3’CCA and guides the ASL into a correct position within the central cleft of Elp3; (iii) tRNA binding displaces the acetyl-CoA blocking loop and could stimulate local structural rearrangement to facilitate acetyl-CoA binding, (iv) acetyl-CoA binding is secured and hydrolysis is initiated only by the correct modifiable substrate tRNAs in analogy to canonical acetyl transferases; (v) the released acetyl group is transported to the SAM domain; (vi) the Fe–S cluster generates a 5’dA-radical; (vii) an acetyl radical is generated via the contact of the acetyl group with the 5’dA-radical (viii) the acetyl radical is transferred onto U_34_ bound in close proximity and the modified tRNA leaves the complex.

Foremost, the presented crystal structures of DmcElp3-DCA and MinElp3 show striking similarities, indicating a common fold and domain architecture of all Elp3 family members. We confirmed the importance of several previously described active site residues for ligand binding and acetyl-CoA hydrolysis activity. Whereas, K266, Q461, and Y517 stabilize the binding of acetyl-CoA during the hydrolysis reaction, K150 might mimic a substrate lysine residue and trigger the acetyl transfer in the direction of the rSAM domain and the bound tRNA substrate. Our high resolution structural information of the unbound and ligand-bound active site of the Elp3 KAT domain might facilitate the design and optimization of highly specific small compound inhibitors as research tools or for therapeutic applications.

In this respect, we established an acetyl-CoA hydrolysis assay, which allows us to quantitatively investigate the enzymatic activity of Elp3 as well as its substrate selectivity. Importantly, the assay format uncouples the KAT-based activity from the reductive SAM cleavage by the Fe–S cluster in the rSAM domain, which would require fully anaerobic reconstitution of the involved Fe–S cluster. We have employed this divide and conquer approach to identify the mechanism of substrate selection and specificity of Elp3 and Elongator. Although, we have repeatedly failed to reproduce a previously described modification assay that was based on isotope labelling^20^, we are able to show that archaeal Elp3 is able to discriminate between modifiable and unmodifiable tRNA substrates at the step of initiating acetyl-CoA hydrolysis.

Notably, the KAT domain of Elp3 responds specifically to tRNA molecules, whereas the addition of previously suspected peptide substrates or other types of nucleic acids does not trigger any detectable enzymatic activity. In particular, no stimulation of acetyl-CoA hydrolysis activity was observed in the case of an isolated eukaryotic Elp3 KAT domain. Although additional peptide binding sites on other eukaryotic Elongator subunits cannot be excluded, it appears, that the exposure of the hypothetical peptide binding site, which is normally blocked by the rSAM domain, does not stimulate a peptide-induced acetyl transfer reaction.

The recognition of substrate tRNAs by Elp3 is independent of other tRNA modifications, but for a subset of tRNA species priming ASL modification might increase the necessary ASL conformation and modification activity. The N-termini of Elp3 proteins seem to contribute to the specific recruitment of mature tRNAs carrying a 3’CCA, promote proper binding of the ASL into the active site and facilitate sequence-specific tRNA selection and specificity by sequence variation in different species. In summary, our data suggest that tRNAs represent the exclusive substrate for the eukaryotic Elongator complex as well as bacterial and archaeal Elp3 proteins, which all represent genuine tRNA editing enzymes.

## Methods

### Protein expression and purification

The MinElp3, CtElp3, ScElp3, and HsElp3 ORFs were synthesized and cloned into pETM30. The respective sequences are listed in Supplementary Table 1. Mutations were introduced using mutagenesis and truncations or N-termini of MinElp3 were made using PCR followed by restriction digestion and insertion of the amplified gene fragments into pETM30 or pETM11. The detailed sequences of the respective primers are listed in Supplementary Table 2. The correct sequences of all constructs were confirmed by sequencing. All plasmids, including the DmcElp3 constructs^21^, were transformed into the strain BL21 (DE3) CodonPlus-RIL competent cells. Transformed cells were grown in TB broth and protein expressions were induced by isopropyl β-D-1-thiogalactopyranoside IPTG (1 mM) for overnight at 18 °C. The cells were collected and lysed in lysis buffer (50 mM HEPES, pH 7.5, 300 mM NaCl, 2 mM DTT, 10% glycerol, 1 mM MgCl_2_, protease inhibitor, lysozyme and DNase). The spun supernatant was obtained by centrifugation at 80,000 × *g* at 4 °C and subjected to a GSTPrep column for protein binding. The target protein was eluted by applying elution buffer containing 50 mM HEPES, pH 7.5, 300 mM NaCl, 2 mM DTT, 10% glycerol and 18 mM GSH. The protein elute was collected and mixed with GST-TEV protease and dialyzed overnight to cleave off the GST tag. The protein solution was then applied back to a GSTPrep column to remove the GST-TEV protease and uncleaved protein. The flow-through was collected and applied to a heparin column and the protein was eluted by applying the gradient elution method (buffer condition: 20 mM HEPES, pH 7.5, 3 M KCl, 2 mM DTT). The protein elute was applied to a size-exclusion chromatography column (S200, 26/60, GE Healthcare) and the buffer was exchanged to 20 mM HEPES, pH 7.5, 150 mM NaCl, 5 mM DTT. The protein concentration was measured and the concentrated purified protein was aliquoted and stored at −80 °C or subjected to crystallization trials. Protein mass of samples was determined using MALS/RALS (Malvern) equipped with a S200 gel filtration column (15/30, GE Healthcare). In the case of His-tagged MinElp3_1–77_ protein, the purification step and conditions were the same as described above but using NiNTA beads for protein binding followed by 250 mM imidazole elution.

### Protein crystallization and structure determination

Purified DmcElp3_390–406(GSGSG)/E386A_ was concentrated to 10 g/L and mixed with 1 mM DCA (Jena Bioscience). The protein solution was combined with equal amount of crystallization buffer (100 mM MES, pH 6.3 and 4% PEG 4000). The purified MinElp3 protein solutions (12 mg/ml) was prepared and crystallized at 22 °C in the condition of 0.1 M phosphate/citrate (pH 3.8), 0.2 M LiSO_4_ and 12% PEG1000 or 0.1 M citrate (pH 3.8), 0.1 M LiCl and 17% PEG6000. All crystals were grown suing hanging-drop vapor-diffusion method. Crystals appeared after 3 days and were cryo-protected at the seventh day in a 30% glycerol solution and flash frozen in liquid nitrogen. Datasets were collected on a Pilatus 6M-F detector at the BESSY II synchrotron (Berlin) for DmcElp3-DCA, MinElp3_Δ1–46_, and MinElp3_Δ1–54_, and DESY synchrotron (Hamburg) for MinElp3_Δ1–19_. Data processing were performed with XDS/XSCALE^61^ and the structures were solved using Phaser^62^ and molecular replacement approach based on the existing DmcElp3 model (5L7L) and further refined using Phenix^63^. The refinement statistics of the respective models are given in Table 1. The geometry and stereochemistry of the models were validated using MolProbity^64^. Structural models and superimpositions were analyzed and generated using Pymol^65^ and Coot^66^. For additional model validation of the presence of DCA, simulated annealing omit maps were calculated using phenix^63,67^. The electrostatic potential was calculated using APBS^66^ and PDB2PQR.

### Peptide synthesis and purification

We synthesized three peptides (H3: YSTGGKAPRK; H4: YRKGGKGLGK; TK40: YMPSDKTIGG) that are known as acetyltransferase substrates^18,50^. We also include an additional tyrosine residue for facilitated concentration determination. All peptides were synthesized as C-terminal amides on a CEM Liberty Blue Microwave Peptide Synthesizer at 0.1 mmol scale by using standard Fmoc-solid phase peptide synthesis methodology on Rink Amide AM Resin (0.9 mmol/g, 100–200 mesh)^68^. As recommended by manufacturer DIC/Oxyma Pure in DMF were used as coupling activators (in fivefold molar excess of Fmoc-AA-OH) and 10% piperazine in NMP/ethanol (9:1) for deprotection. Following the final deprotection, N-terminal acetylation was performed with 10% acetic anhydride in DMF. Cleavage was performed for 3 h at room temperature with TFA/H_2_O/TIS (94/3/3). Following cleavage TFA solution was concentrated under nitrogen stream for 30 min. The concentrated peptide solution was triturated with cold diethyl ether and the precipitate was centrifuged. Precipitated peptide was washed with diethyl ether and ethyl acetate (twice) and dried under vacuum overnight. Crude peptide was dissolved in 6 M urea and purified by semipreparative RP-HPLC on C18 column. The purified fractions were collected, frozen, and lyophilized. Purified peptides were analyzed on analytical RP-HPLC and confirmed by electrospray ionization mass spectrometry (ESI-MS). Peptides labeling with fluorescence probe was carried out after the solid phase peptide synthesis on the resin. In brief, dye conjugation was performed after coupling and deprotection of the last amino acid on the fully protected (except the N-terminal amino group) peptide while still attached to the resin. Peptide resin (15 µmols) slurry was prepared in 100 µl anhydrous DMF (VWR Chemicals). Cyanine5 NHS ester (Abcam) was dissolved in anhydrous DMSO (Sigma) and it was added to peptide-resin slurry at equal molar ratio and the incubation was carried out at room temperature for 2 h. i-Pr2NEt - N, N-Diisopropylethylamine (DIPEA, 50 mM) was then added dropwise to the slurry solution to adjust pH to 8.5 and followed by stirring overnight. The Cy5-peptide-resin was extensively washed with several solutions as follows: DMF, DCM, isopropanol, ethyl acetate, and dried under vacuum. Cleavage protocol and all post synthetic steps were the same as previously described procedure for non-labeled peptides. Cy5-labeled peptides were further purified using HPLC chromatography on the RP C18 column. Purified Cy5-peptides were lyophilized and stored at −80 °C.

### Yeast culture and bulk tRNA purification

The two *S. cerevisiae* strains (UMY2893 and UMY2916)^69^ were cultured from a single colony at 30 °C for overnight with agitating at 200 rpm. The cells (1L, OD_600_ at 0.2) were then further cultured until OD_600_ at 1.5 and followed by centrifugation at 3000 × *g* at 4 °C for 10 min to collect cells. The cells were then washed with H_2_O and resuspended in 30 ml TE buffer (10 mM Tris-HCl, pH 7.5, 1 mM EDTA) with addition of 0.1 M NaOAc and 1% SDS. A cryo-mill operated with liquid nitrogen was used to grind and lyse cells. The powder was thawed on ice and followed by adding 30 ml TRIzol (Thermo Fisher Scientific). The solution was vortexed for 1 min and incubated at RT for 20 min. The RNA was purified by chloroform extraction and followed by two step precipitations in 8 M LiCl and followed by 0.3 M NaOAc and 75% EtOH precipitation at −20 °C for overnight. The RNA was spun into a pellet by centrifugation at 4000 × *g* at 4 °C for 30 min. The pellet was washed in 75% EtOH for three times, air-dried and resuspended in 3 ml 10 mM NaOAc pH 5.2. The RNA was then further applied to a S200 gel filtration column (HiLoad 26/600) equilibrated in 10 mM NaOAc (pH 5.2) to purify tRNAs. The tRNA fractions were pooled and tRNA was precipitated using the method described above and resuspended in 10 mM NaOAc (pH 5.2) for storage.

### Acetyl-CoA hydrolysis

Proteins (10 μM) were mixed with 100 μM acetyl-CoA and tRNAs (2 μM) or peptides (2 μM) and incubated at 37 °C for 20 min. The reaction was applied to a 3 kDa cutoff concentrator (EMD Millipore) to remove proteins. The flow through (50 μL) was collected and transferred to a 96-well plate. Acetyl-CoA quantity was determined using an acetyl-CoA assay kit (Sigma) in accordance to manufacturer’s instructions. An end-point assay was performed and the fluorescence intensity (*λ*_Excitation_ 535 nm; *λ*_Emission_ 587 nm) was measured using a plate reader (TECAN); *n* = 3.

### tRNA constructs and in vitro transcription and purification

All tRNA genes were amplified by PCR using genomic DNA from yeast as template and the primers that contain the complementary sequences to pUC19. Restriction free cloning method was performed to construct all amplified tRNA genes downstream of a T7 promoter. Downstream of every gene a BpiI cutting site was introduced, which is used to linearize the DNA for run off T7 RNA polymerase-driven in vitro transcription. The addition of CCA to the 3′-end of tRNA genes was performed by simple mutagenesis of the template construct. For in vitro transcription (250 μL reaction), the reaction contained linear DNA template (20 ng), 8 mM of each NTPs (ATP, GTP, and UTP), 6 mM of CTP and 2 mM of Cy5-lableled CTP, T7 RNA polymerase (0.5 g/L), RNasin (0.025 g/L), MgCl_2_ (2~8 mM), pyrophosphatase (1 U) and the reaction buffer (40 mM Tris, pH 8.0, 5 mM DTT, 1 mM spermidine, and 0.01% Triton X-100). All the ingredients were mixed at RT first followed by the incubation at 37 °C for overnight. The next day, the RNase free DNase (2U) was added to the reaction for further incubation at 37 °C for 15 min. The reaction was then stopped by the addition of EDTA (50 mM). The transcribed tRNA was purified using a DEAE column directly^70^ and eluted tRNA fractions were pooled and precipitated in EtOH at −80 °C for overnight. The tRNA containing solution was subjected to centrifuge at 20,000 × *g* at 4 °C for 30 min. The pellet was then washed in 70% EtOH, air-dried and re-dissolved in annealing buffer (20 mM HEPES, pH 7.5, 50 mM NaCl, and 50 mM KCl). Cy5-labeled tRNA was first renatured by heating in the annealing buffer at 80 °C for 2 min and slowly cooled to 60 °C using a thermo-cycler. MgCl_2_ was added to tRNA and the procedure continued by cooling down to 25 °C. Annealed tRNAs were further purified by applying them to a gel filtration column (S75, 10/30) equilibrated in 20 mM HEPES, pH 7.5, 150 mM NaCl, 5 mM DTT, and 1 mM MgCl_2_. The purified tRNAs were then stored at −80 °C.

### EMSA

Cy5-labeled RNA oligos (ASL yeast tRNA^Ser^_UGA_ 5′-GACAGACUUGAAAUCUGUU-3′ and polyU 5′-UUUUUUUUU-3′) were purchased from Sigma. Proteins were serially diluted, and Cy5-labeled RNA (~1 ng/lane) as well as Cy5-labeled tRNA were added for incubation at 25 °C for 30 min. The RNA-protein complex and free RNA were resolved by 6% native gel and separated by running for 6 h at 100 V at 4 °C. The gels were scanned using Typhoon (GE Healthcare) and the images were analyzed and viewed by ImageQuant (GE Healthcare).

### Microscale thermophoresis

MinElp3 (15 g/l) or DmcElp3 (10 g/l) were prepared in twofold serial dilutions in PCR tubes. The Cy5-labeled tRNA (30 nM) or Cy-labeled H3 peptide (10 nM) was mixed with diluted proteins with equal amount for incubation at 25 °C for 15 min. The samples were subjected to premium capillary tubes and analyzed using MO. CONTROL (Nanotemper) with the temperature setting at 25 °C. The *K*_d_s were calculated based on triplicate measurement using MO. Affinity software (Nanotemper), *n* = 3.

### Isothermal titration calorimetry measurements

ITC was performed with Nano ITC 2G (TA Instruments). To measure the MinElp3 interaction with acetyl-CoA, histone 3 peptide or tubulin peptide, proteins were dialyzed against the ITC buffer (20 mM HEPES, pH 7.5, 150 mM NaCl, and 1 mM β-mercaptoethanol) extensively overnight at 4 °C. The reaction cell was filled with proteins (10–12 µM); whereas, the ligands were prepared in the same ITC buffer at 2.47 mM (except H3 was 1.6 mM) and loaded into a dosing syringe. Each injection (10 µl) was performed in 300 s intervals. All experiments were conducted at 25 °C with a stirring rate of 250 rpm. The results were analyzed using NanoAnalyze software (TA instruments).

## Supplementary information

Supplementary Information

Source Data

Reporting Summary

## Data Availability

Data generated in this publication are available from corresponding author on reasonable request. The atomic coordinates and respective structure factors for DmcElp3-DCA (PDB ID 6IA6), MinElp3Δ1-19 (PDB ID 6IA8), MinElp3Δ1-46 (PDB ID 6IAZ) and MinElp3Δ1-54 (PDB ID 6IAD) have been validated and deposited at the European Protein Data Bank. The source data underlying Figs 3, 4, Supplementary Fig. 1, 2, 3, 4, 5 and the numerical values of the graphs, including Figs 2, 3, 4 and Supplementary Fig. 3, 4, 5 and 6 are provided as a Source Data file.
