# Supplementary material for: The Elongator subunit Elp3 is a non-canonical tRNA acetyltransferase
Source: Nat Commun. 2019 Feb 7;10:625. doi: 10.1038/s41467-019-08579-2 (PMC6367351; doi:10.1038/s41467-019-08579-2)
Supplement: Supplementary file 1 — Supplementary Information [file 41467_2019_8579_MOESM1_ESM.pdf]

Supplementary Information

The Elongator subunit Elp3 is a non-canonical tRNA acetyltransferase

Ting-Yu Lin et al.

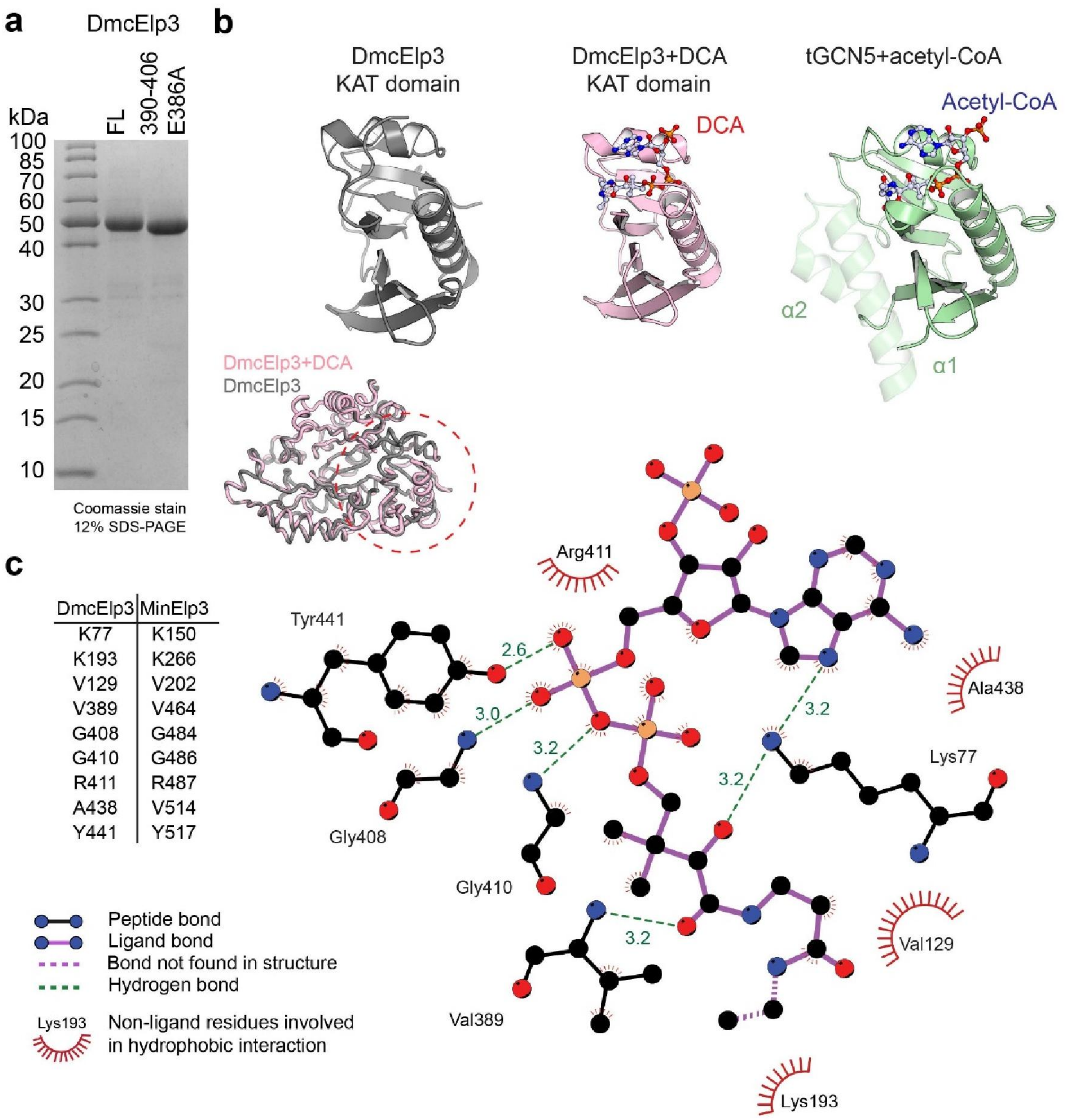

Supplementary Figure 1. Illustrations of DCA bound to DmcElp3$_{390\text{-}406(GSGSG)/E386A}$. (**a**) SDS-PAGE of aerobically purified DmcElp3s. (**b**) Superposition of DmcElp3 (5L7J) and DmcElp3$_{390\text{-}406(GSGSG)/E386A}$ in the presence of DCA shown in cartoon tube representation. The conserved KAT domain is highlighted in dotted red circle. Structural comparison of the KAT domains of DmcElp3 and tGCN5 in the complex with acetyl-CoA. (**c**) LigPlot analysis of DCA interaction with DmcElp3$_{390\text{-}406(GSGSG)/E386A}$. Carbon (black), nitrogen (grey blue), oxygen (red), phosphate (light brown). Equivalent residue numbers in DmcElp3 and MinElp3 are given on the left. Source data are provided as a Source Data file.

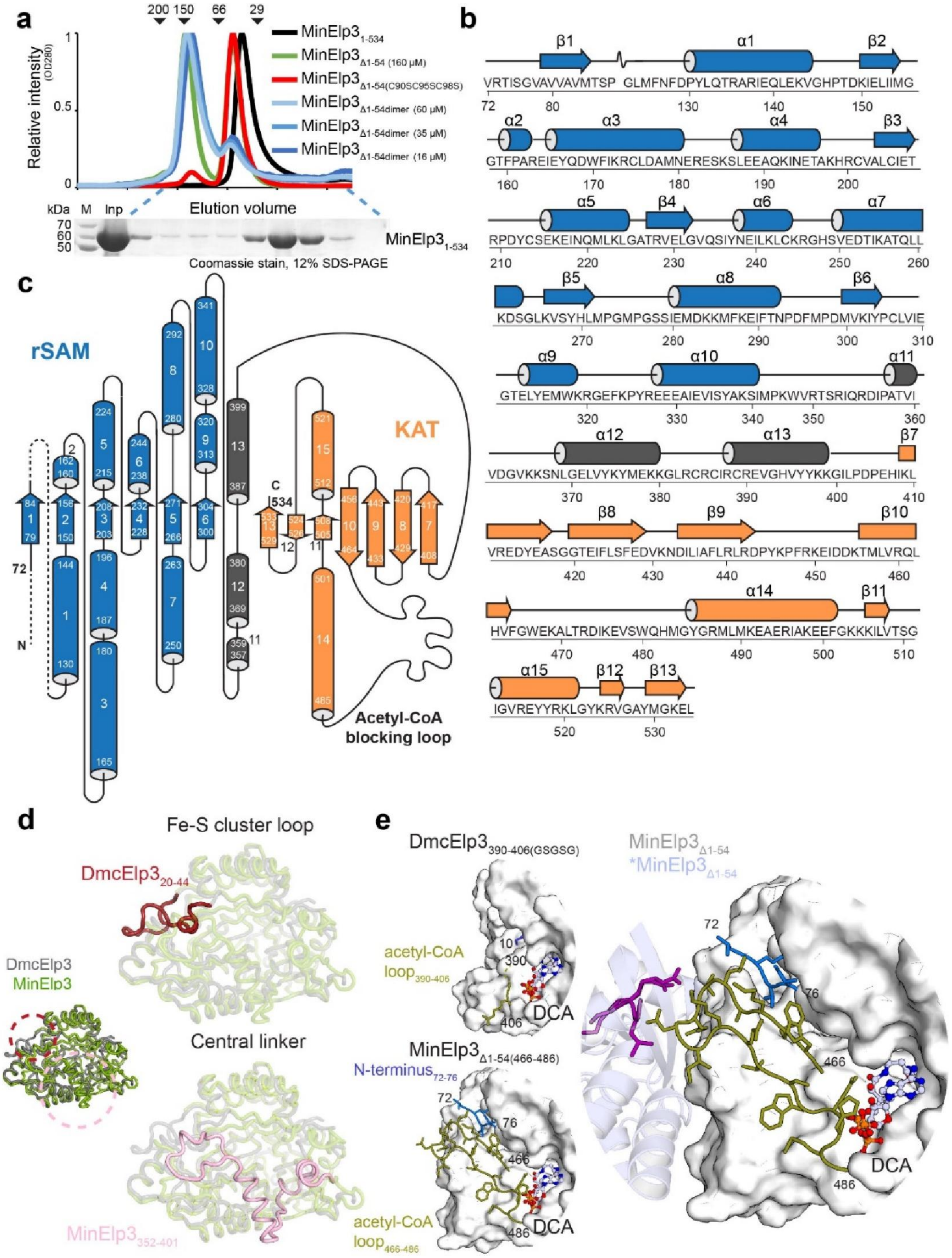

Supplementary Figure 2. Topology and structural features of MinElp3. (**a**) S200 gel filtration profile of different MinElp3s and their concentrations are indicated otherwise are 160 µM. The eluted fractions were resolved in a 12% SDS-PAGE and visualized by Coomassie staining. (**b**) MinElp3 protein sequence and its secondary structure elements are presented in the structural model. (**c**) Topology of MinElp3 indicating secondary structure elements. (**d**) Superposition of the apo DmcElp3 (left) and the apo MinElp3$_{\Delta 1\text{-}54}$ in cartoon tube presentation while the Fe-S cluster region of DmcElp3 (top) and the central linker region of MinElp3 (bottom) are highlighted separately. (**e**) Superpositions of the MinElp3 and DCA (bottom left) in the reference to DmcElp3-DCA structure (top left). The interaction of MinElp3 with its symmetry mate (*MinElp3$_{\Delta 1\text{-}54}$) (right). Source data are provided as a Source Data file.

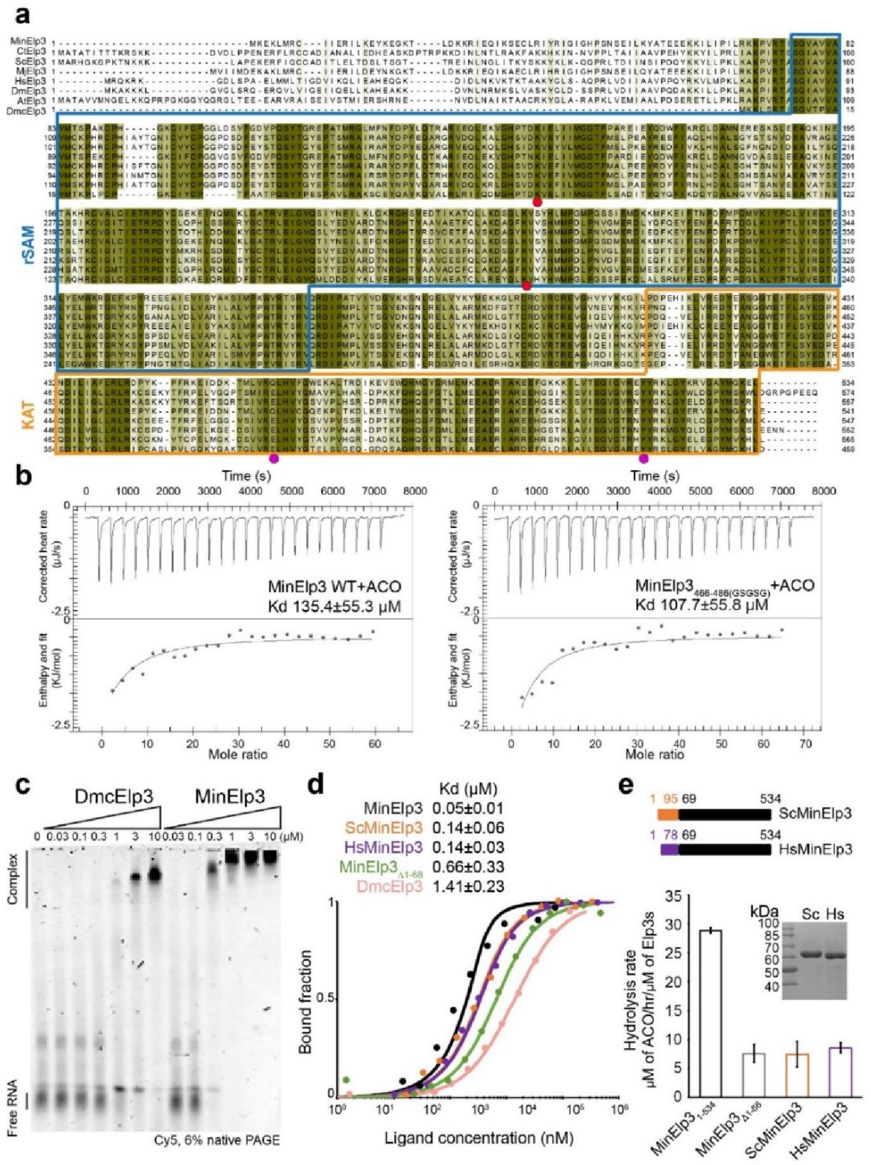

Supplementary Figure 3. The different features of Elp3s in sequence and tRNA binding and acetyl-CoA affinity. (**a**) Sequence alignment of selected Elp3s using Jalview. *D. mccartyi* (DmcElp3) (YP307690), *M. infernus* (MinElp3) (YP003616086), *M. jannaschii* (MjElp3) (AAB99138), *S. cerevisiae* (ScElp3) (NP015239), *D. melanogaster* (DmElp3) (NP608834), *A. thaliana* (AtElp3) (NP568725), *H. sapiens* (HsElp3) (NP060561), *C. thermophilum* (CtElp3) (XP_006697164.1). The conserved amino acids (score above 1.5) are indicated in gradient green depending on the conservation scores. rSAM and KAT domains are highlighted in blue and orange boxes, respectively. K150 and K266 are highlighted by red circles while the Q461 and Y541 are indicated by purple circles. (**b**) ITC measurements of the interaction of acetyl-CoA with MinElp3 or MinElp3$_{466-486(GSGSG)}$. The K_ds are indicated. (**c**) EMSA analysis of Elp3s binding to(Dmc)tRNA$^{Glu}_{UUC}$(CCA). The free tRNA and protein-tRNA complex are labelled. (**d**) MST analyses of Elp3s binding to (Dmc)tRNA$^{Glu}_{UUC}$(CCA) and the calculated Kds are indicated, n=3. (**e**) The N-terminus of MinElp3 (aa 1-69) was replaced with the N-terminus from either ScElp3 (aa 1-95) or HsElp3 (aa 1-78). The purified chimeric proteins were analyzed using SDS-PAGE (inset) and tested for acetyl-CoA hydrolysis. Data represent mean ± standard error of the mean (SEM). Source data are provided as a Source Data file.

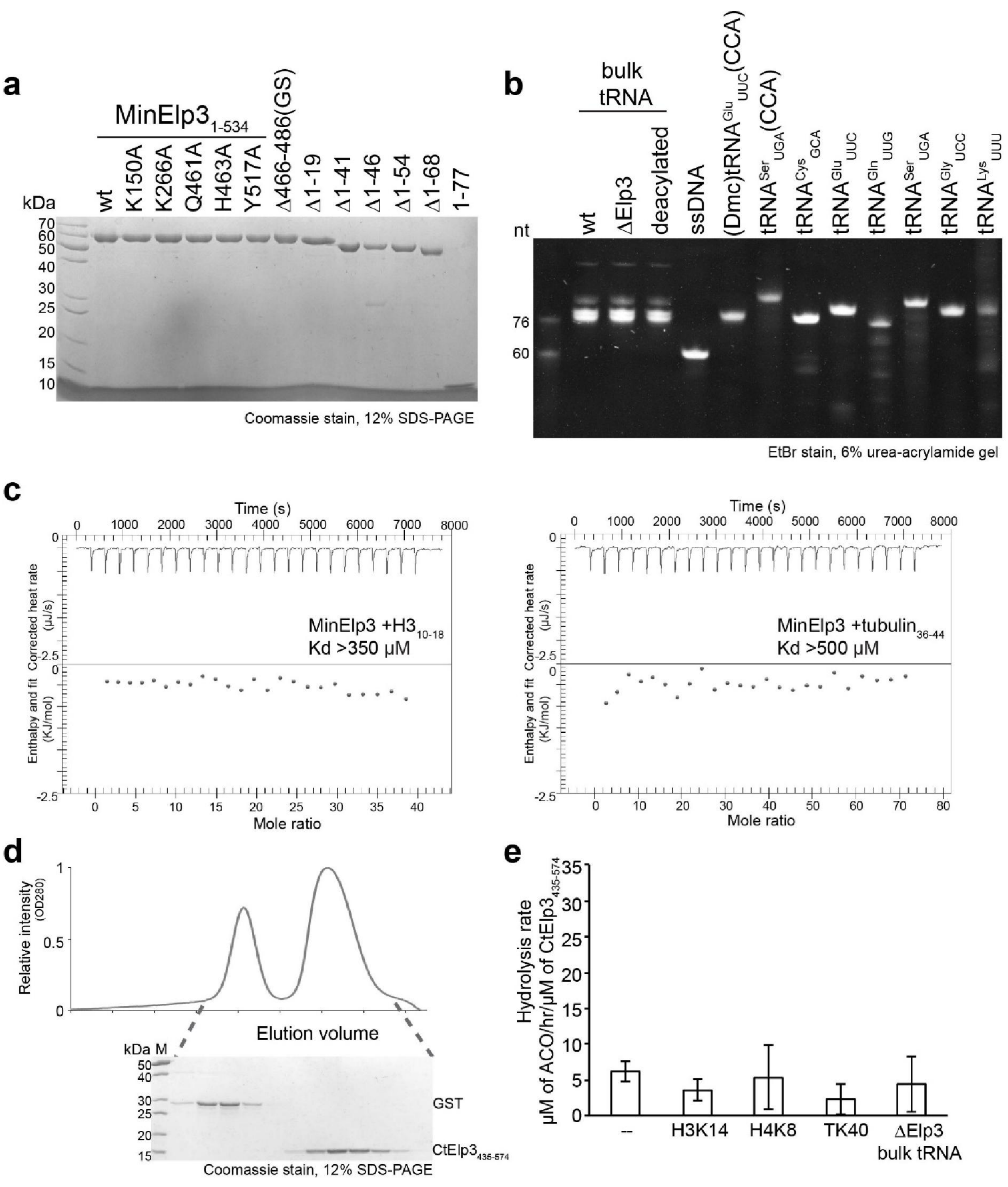

Supplementary Figure 4. Lysine-containing peptides do not interact with MinElp3 and not trigger activated acetyl-CoA hydrolysis. (**a**) SDS-PAGE of aerobically purified MinElp3s. (**b**) Urea gel of yeast bulk tRNAs, ssDNA and *in vitro* transcribed tRNAs. (**c**) ITC measurements of the interaction of peptides with MinElp3. (**d**) S200 gel filtration profile of the aerobically purified CtElp3$_{435-574}$. The eluted fractions were resolved in a 12% SDS-PAGE and visualized using Coomassie stain. CtElp3$_{435-574}$ containing fraction were pooled and used for subsequent experiments. (**e**) Acetyl-CoA hydrolysis activity of CtElp3$_{435-574}$ in the presence of lysine-containing peptides or yeast bulk tRNA. Data represent mean ± SEM. Source data are provided as a Source Data file.

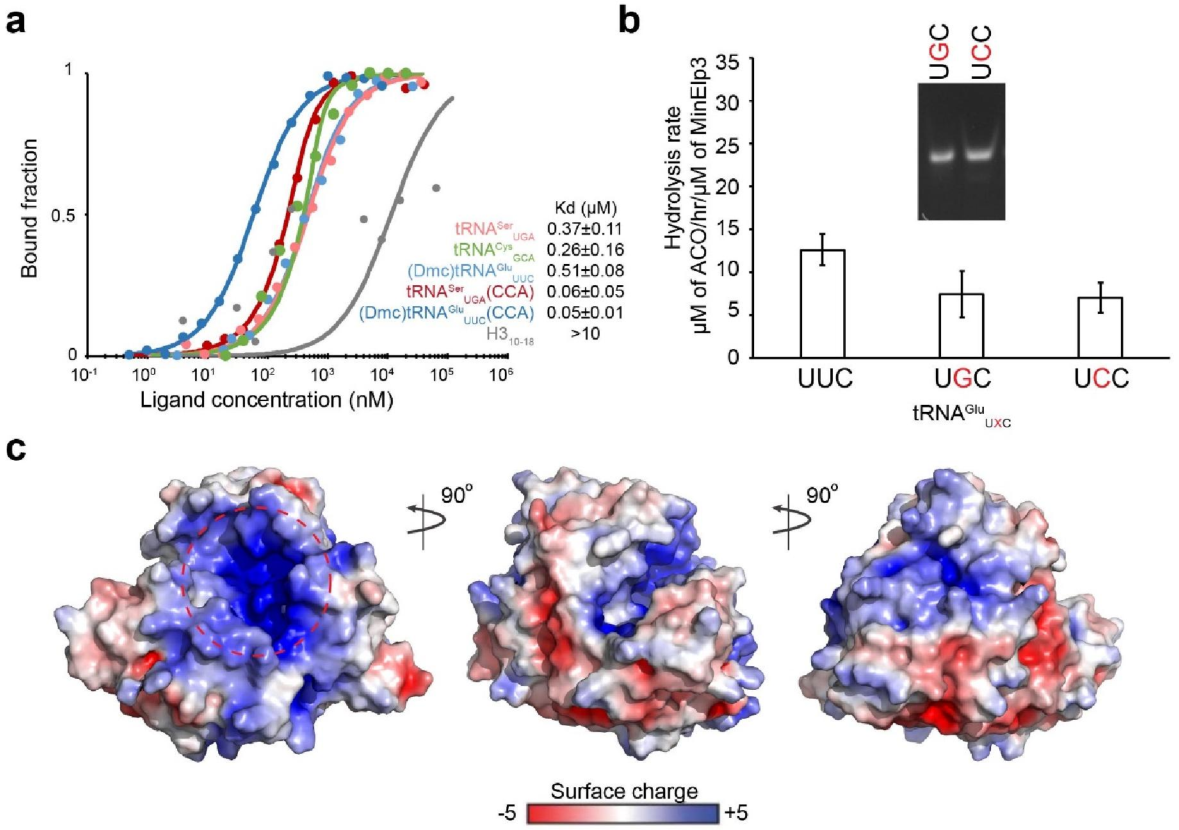

Supplementary Figure 5. The key feature tRNA for activated acetyl-CoA hydrolysis of Elp3. (**a**) MST analyses of MinElp3 binding to different tRNA molecules. The K_ds are presented in the inset. n=3. (**b**) Acetyl-CoA hydrolysis activity of MinElp3 in the presence of yeast $tRNA^{Glu}_{UUC}$ (wt) and the two U_{35} substituted mutated tRNAs. The urea gel of the two mutated tRNAs are shown in the inset. (**c**) Surface representation of surface charge distribution of MinElp3 from APBS analysis. The conserved basic tRNA binding site is indicated in with a red dotted circle. Data represent mean ± SEM. Source data are provided as a Source Data file.

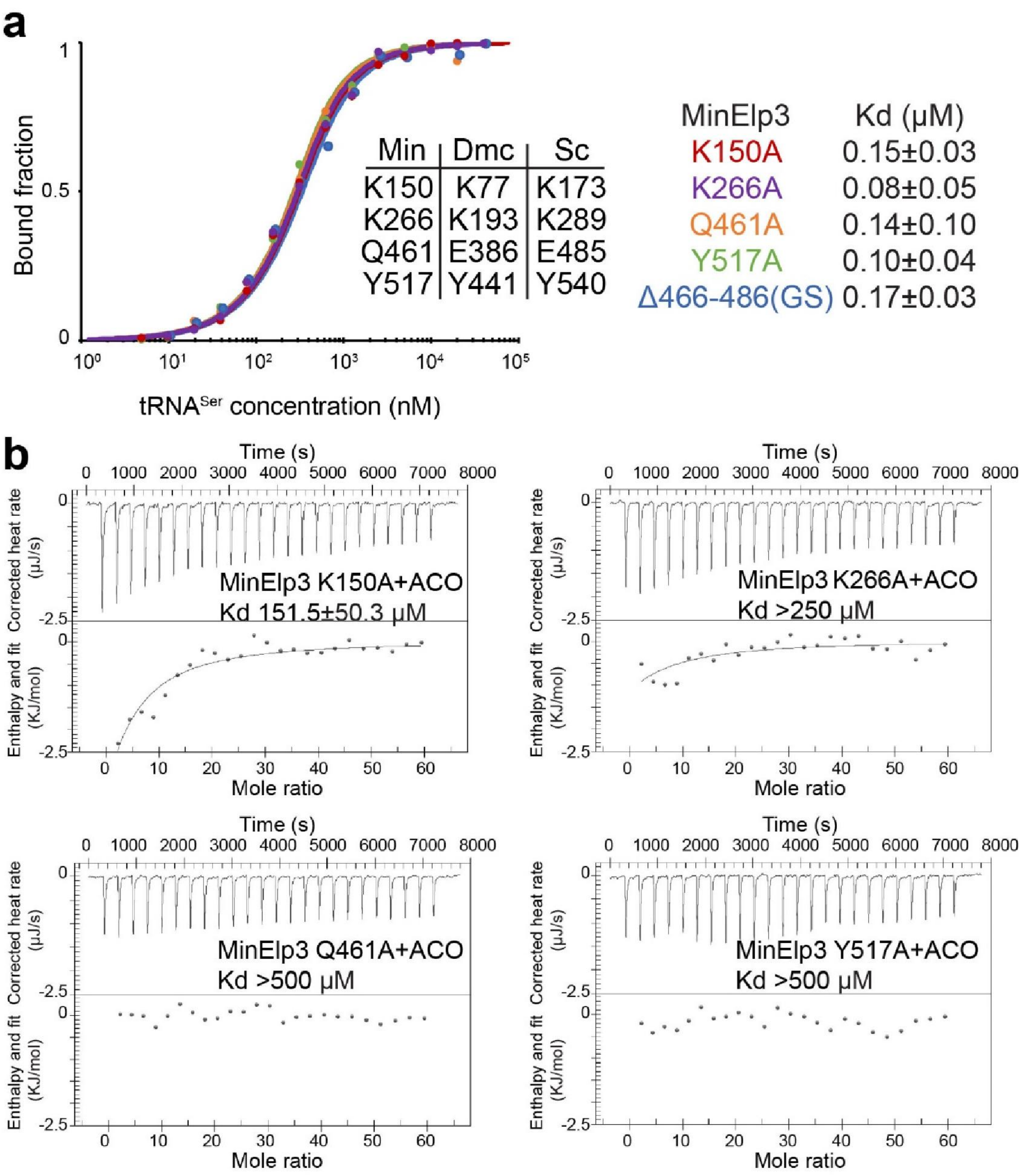

Supplementary Figure 6. The active site mutants interact with tRNA indifferently but with acetyl-CoA at different levels. (**a**) MST analyses of MinElp3s binding to yeast $tRNA^{Ser}$. The K_ds are presented in the inset and the equivalent amino acid residues in DmcElp3 and ScElp3 are presented. n=3. (**b**) ITC measurements of the interaction of acetyl-CoA with MinElp3s. The K_ds are indicated. Data represent mean ± SEM. Source data are provided as a Source Data file.

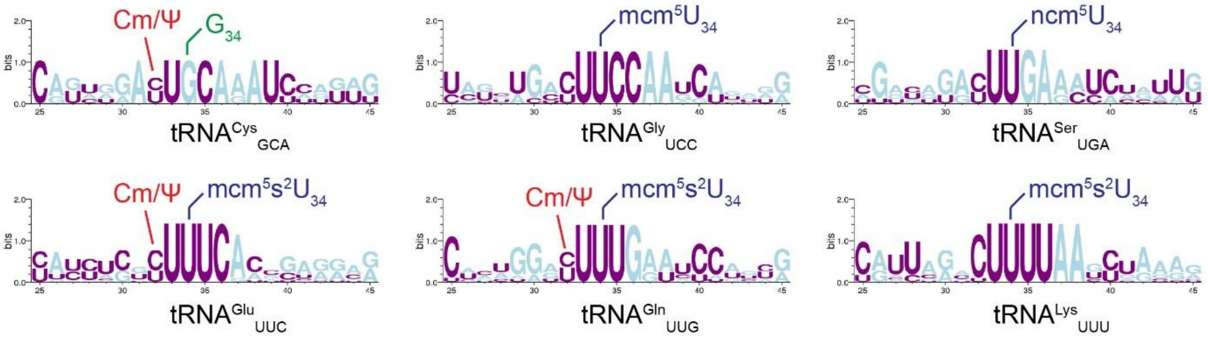

Supplementary Figure 7. Analysis of sequence conservation in the ASL region of yeast tRNAs. All gene sequences, including $tRNA^{Cys}_{CGA}$ (4), $tRNA^{Gly}_{UUC}$ (7), $tRNA^{Ser}_{UGA}$ (8), $tRNA^{Lys}_{UUU}$ (11), $tRNA^{Glu}_{UUC}$ (11), $tRNA^{Gln}_{UUG}$ (10), and were retrieved from http://gtrnadb.ucsc.edu/[1], analyzed and displayed using weblogo 3 (http://weblogo.berkeley.edu/)[2,3]. Modified bases 32 and 34 are indicated.

Supplementary Table 1. Sequences of ORFs

MinElp3	ATGGCGAAAGAAAAGCTGATGCGTTGCATCATCGAGCGTATCCTGAAAGAGTACAAG<br>GAAGGTAAAACCCTGGACAAGAAACGTATCGAACAGATCAAGAGCGAGTGCCTGCGT<br>ATCTACCGTATCGGTATCGGCCACCCGAGCAACAGCGAGATCCTGAAATACGCGACCG<br>AGGAAGAGAAGAAAATCCTGATCCCGATCCTGCGTAAGAAACCGGTGCGTACCATCA<br>GCGGCGTGGCTGTGGTTGCTGTTATGACCAGCCCGGCTAAGTGCCCGCACGGTAAAT<br>GCATCTTCTGCCCGGGTGGCCTGGACAGCGTGTTCGGTGACGTTCCGCAAAGCTACAC<br>CGGTCGTGAACCGGCTACCATGCGTGGTCTGATGTTCAACTTCGACCCGTACCTGCAG<br>ACCCGTGCTCGTATCGAGCAGCTGGAAAAAGTGGGTCACCCGACCGATAAAATCGAG<br>CTGATCATCATGGGTGGCACCTTCCCGGCTCGTGAGATCGAATACCAGGACTGGTTCA<br>TCAAGCGTTGCCTGGATGCGATGAACGAGCGTGAAAGCAAAAGCCTGGAAGAGGCTC<br>AGAAGATCAACGAAACCGCGAAACACCGTTGCGTGGCTCTGTGCATCGAAACCCGTCC<br>GGACTACTGCAGCGAGAAGGAAATCAACCAGATGCTGAAACTGGGTGCGACCCGTGT<br>GGAACTGGGCGTTCAGAGCATCTACAACGAGATCCTGAAGCTGTGCAAACGTGGCCA<br>CAGCGTGGAAGACACCATCAAGGCTACCCAGCTGCTGAAGGATAGCGGTCTGAAAGT<br>TAGCTACCACCTGATGCCGGGTATGCCGGGCAGCAGCATCGAAATGGACAAGAAAAT<br>GTTCAAGGAGATCTTCACCAACCCGGACTTCATGCCGGATATGGTGAAAATCTACCCG<br>TGCCTGGTTATCGAGGGCACCGAACTGTACGAGATGTGGAAGCGTGGTGAATTCAAA<br>CCGTACCGTGAAGAGGAAGCGATCGAAGTGATCAGCTACGCTAAGAGCATCATGCCG<br>AAATGGGTTCGTACCAGCCGTATCCAGCGTGACATCCCGGCGACCGTGATCGTTGATG<br>GTGTGAAGAAAAGCAACCTGGGCGAACTGGTTTACAAGTACATGGAGAAGAAAGGCC<br>TGCGTTGCCGTTGCATCCGTTGCCGTGAAGTGGGTCACGTTTACTACAAGAAAGGCAT<br>CCTGCCGGACCCGGAGCACATCAAGCTGGTGCGTGAAGATTACGAGGCGAGCGGTGG<br>CACCGAAATCTTCCTGAGCTTCGAGGACGTTAAGAACGATATCCTGATCGCTTTCCTGC<br>GTCTGCGTGACCCGTACAAGCCGTTCCGTAAAGAGATCGACGATAAGACCATGCTGGT<br>GCGTCAGCTGCACGTTTTCGGTTGGGAAAAGGCGCTGACCCGTGATATCAAAGAGGT<br>GAGCTGGCAGCACATGGGTTACGGCCGTATGCTGATGAAGGAAGCGGAACGTATCGC<br>TAAAGAGGAATTCGGCAAGAAAAAGATCCTGGTGACCAGCGGTATCGGCGTTCGTGA<br>ATACTACCGTAAGCTGGGTTACAAACGTGTTGGTGCTTACATGGGCAAGGAGCTGTAA
ScElp3 (N-terminus aa 1-95)	ATGGCGGCTCGTCATGGAAAAGGCCCAAAAACTAACAAAAAAAAGCTAGCACCTGAA<br>AAGGAAAGGTTTATACAATGTTGTGCTGATATCACATTAGAGTTAACAGATTCTTTAAC<br>CTCGGGAACAACAAGAGAAATTAATCTGAATGGTTTGATTACTAAATATTCAAAGAAA<br>TATAAACTAAAGCAACAACCAAGGTTAACCGATATCATTAATTCTATTCCAGACCAATA<br>CAAAAAATATTTATTACCCAAATTGAAGGCTAAGCCAGTAAGAACAGCATCGGGT
HsElp3 (N-terminus aa 1-78)	ATGGCGCGTCAGAAGCGTAAAGGCGATCTGAGCCCGGCTGAGCTGATGATGCTGACC<br>ATCGGTGACGTGATCAAGCAGCTGATCGAGGCGCACGAACAGGGCAAAGACATCGAT<br>CTGAACAAGGTTAAAACCAAGACCGCGGCTAAGTACGGTCTGAGCGCTCAGCCGCGT<br>CTGGTGGACATCATCGCGGCTGTTCCGCCGCAGTACCGTAAAGTGCTGATGCCGAAAC<br>TGAAGGCG
CtElp3 (aa 435-574)	AACCGAATTGAGCTTGTGCGCCGTGATTACACGGCCAACGGAGGTTGGGAGACCTTCC<br>TTGCTTACGAAGATCCTAAGCAAGACATCTTGATTGCTCTGCTTCGCCTCCGAAAGTGC<br>AGCGAAAAGTATACGTTCCGTCCAGAGCTGGTCGGGCAGCCCACTAGCATGATACGT<br>GAGCTTCACGTGTATGGTATGGCGGTCCCGCTTCATGCACGTGATCCTAAGAAGTTCC<br>AGCATCAAGGGTTTGGTACGCTATTGATGGAGGAGGCAGAGCGAATTGCACGAGAAG<br>AGCATGGAAGTATCAAAATCAGCGTCATCTCCGGCGTGGGCGTTAGAAGTTATTATCG<br>CAAACTGGGGTATTGGCTTGACGGACCGTACATGTCCAAGTGGTTAGATGGCCGACCA<br>GGGCCTGAAGAACAATAA

Supplementary Table 2. Primer sequences

Primer name	Primer sequence (5’->3’)
DmcElp3_Δ390-406(GS)_F	CGGCAGCGGTAGGGGTCTTGGACGG
DmcElp3_Δ390-406(GS)_R	CCGTCCAAGACCCCTACCGCTGCCG
DmcElp3_E386A_F	ACCCACATGCAGTGCACGCACCAGCCC
DmcElp3_E386A_R	GGGCTGGTGCGTGCACTGCATGTGGGT
DmcElp3_H388A_F	CTGCCGCTACCCACAGCCAGTTCACGCACCAG
DmcElp3_H388A_R	CTGGTGCGTGAACTGGCTGTGGGTAGCGGCAG
DmcElp3_R411T_F	CCGCCAGCAGTTTCGTTCCAAGACCCCGGT
DmcElp3_R411T_R	ACCGGGGTCTTGGAACGAAACTGCTGGCGG
MinElp3_Δ1-19_F	TCCAAGCCATGGCGGGTAAAACCCTGGACAAGAAACG
MinElp3_Δ1-41_F	TCCAAGCCATGGCGGGTATCGGCCACCCGAG
MinElp3_Δ1-46_F	TCCAAGCCATGGCGAGCAACAGCGAGATCCTG
MinElp3_Δ1-54_F	TCCAAGCCATGGCGACCGAGGAAGAGAAG
MinElp3_Δ1-68_F	TCCAAGCCATGGCGAAGAAACCGGTGCGTACC
MinElp3_R	TCCAAGCTCGAGGGTACCTTATTACAGC
MinElp3_Δ466-486(GS)_F	GCGTCAGCTGCACGTTTTCGGTAGCGGCAGCGGTCGTATGCTGATGAAG GAAGCG
MinElp3_Δ466-486(GS)_R	CGCTTCCTTCATCAGCATACGACCGCTGCCGCTACCGAAAACGTGCAGCT GACGC
MinElp3_C90SC95SC98S_F	ATGACCAGCCCGGCTAAGAGCCCGCACGGTAAAAGCATCTTCAGCCCGG GTGGCCTGGAC
MinElp3_C90SC95SC98S_R	GTCCAGGCCACCCGGGCTGAAGATGCTTTTACCGTGCGGGCTCTTAGCC GGGCTGGTCAT
MinElp3_K150_F	ATGATGATCAGCTCGATTGCATCGGTCGGGTGACCCAC
MinElp3_K150_R	GTGGGTCACCCGACCGATGCAATCGAGCTGATCATCAT
MinElp3_K266_F	GCTGAAGGATAGCGGTCTGGCAGTTAGCTACCACCTGATG
MinElp3_K266_R	CATCAGGTGGTAGCTAACTGCCAGACCGCTATCCTTCAGC
MinElp3_Q461A-F	AAAACGTGCAGCGCACGCACCAGCATGGTCTTATCG
MinElp3_Q461A-R	CGATAAGACCATGCTGGTGCGTGCGCTGCACGTTTT
MinElp3_H463_F	TCCCAACCGAAAACGGCCAGCTGACGCACCAG
MinElp3_H463_R	CTGGTGCGTCAGCTGGCCGTTTTCGGTTGGGA
MinElp3_Y517A_F	TAACCCAGCTTACGGTAGGCTTCACGAACGCCGATACC
MinElp3_Y517A_R	GGTATCGGCGTTCGTGAAGCCTACCGTAAGCTGGGTTA
HsElp3_F	TCCAAGCCATGGCGCGTCAGAAGC
HsMinElp3(78-69)_F	CTGATGCCGAAACTGAAGGCGAAGAAACCGGTGCGTACCATC
HsMinElp3(78-69)_R	GATGGTACGCACCGGTTTCTTCGCCTTCAGTTTCGGCATCAG
ScElp3_F	AGGGCGCCATGGCGGCTCGTCATGGAAAAGGCC
ScMinElp3(95-69)_F	CCAGTAAGAACAGCATCGGGTAAGAAACCGGTGCGTACCATC
ScMinElp3(95-69)_R	GATGGTACGCACCGGTTTCTTACCCGATGCTGTTCTTACTGG
MinElp3_F	TCCAAGCCATGGCGAAAGAAAAGCTGATGCGT
MinElp3_1-77_R	TCCAAGCTCGAGTTAGCCGCTGATGGTACGCAC
CtElp3_KAT_F	ATGGCAAACCGAATTGAGCTTGTGCGC
CtElp3_R	TCCAAGCTCGAGTTATTGTTCTTCAGGCCCTGGTCG
Sc_tRNA_Ser_F	CAGATTAATACGACTCACTATAGGCACTATGGCCGAGTGG
Sc_tRNA_Ser_R	CGACCGAATACACCCATGGGAAGACGCCGACACCAGCAGGATTTGAAC
Sc_tRNA_Gly_F	CAGATTAATACGACTCACTATAGGGCGGTTAGTGTAGTGGTT
Sc_tRNA_Gly_R	CGACCGAATACACCCATGGGAAGACGCTGAGCGGTACGAGAATCGAAC
Sc_tRNA_Lys_F	CAGATTAATACGACTCACTATATCCTTGTTAGCTCAGTTGGTAG

Sc_tRNA_Lys_R	CGACCGAATACACCCATGGGAAGACGCCTCCTCATAGGGGGCTCG
Sc_tRNA_Gln_F	CAGATTAATACGACTCACTATAGCTCGTATGGCGCAGTGG
Sc_tRNA_Gln_R	CGACCGAATACACCCATGGGAAGACGCAGGTCTTACCCGGATTCGAAC
Sc_tRNA_Glu_F	CAGATTAATACGACTCACTATATCCGATATAGTGTAACGGCTATC
Sc_tRNA_Glu_R	CGACCGAATACACCCATGGGAAGACGCCTCCGATACGGGGAGTCG
Sc_tRNA_Cys_F	CAGATTAATACGACTCACTATAGCTCGTATGGCGCAGTGG
Sc_tRNA_Cys_R	CGACCGAATACACCCATGGGAAGACGCAGCTCGCACTCAGGATCGAA
Sc_tRNA_Glu(G35)_F	TCTCCACGGTGCAAGCGTGATGTGATAGCCGT
Sc_tRNA_Glu(G35)_R	ACGGCTATCACATCACGCTTGCACCGTGGAGA
Sc_tRNA_Glu(C35)_F	TCTCCACGGTGGAAGCGTGATGTGATAGCCGT
Sc_tRNA_Glu(C35)_R	ACGGCTATCACATCACGCTTCCACCGTGGAGA

Supplementary References

1. Chan, P.P. & Lowe, T.M. GtRNAdb: a database of transfer RNA genes detected in genomic sequence. *Nucleic Acids Res* **37**, D93-7 (2009).
2. Crooks, G.E., Hon, G., Chandonia, J.M. & Brenner, S.E. WebLogo: a sequence logo generator. *Genome Res* **14**, 1188-90 (2004).
3. Schneider, T.D. & Stephens, R.M. Sequence logos: a new way to display consensus sequences. *Nucleic Acids Res* **18**, 6097-100 (1990).
